# MOF-Derived N-Doped Carbon Nanotube-Confined Ni Nanoparticles for the Simultaneous Electrochemical Detection of Cu²⁺ and Hg²⁺ with High Sensitivity and Stability

**DOI:** 10.3390/molecules30051078

**Published:** 2025-02-26

**Authors:** Jiapeng Li, Lili Chen, Yiming Qiao, Li Li, Xin Li, Linbo Deng, Xuemin Duan, Hui Chen, Yansha Gao

**Affiliations:** 1 Jiangxi Provincial Key Laboratory of Drug Design and Evaluation, Flexible Electronics Innovation Institute (FEII), School of Pharmacy, Jiangxi Science and Technology Normal University, Nanchang 330013, China; 13133270915@163.com (J.L.); 19314658155@163.com (L.C.); 13273929093@163.com (Y.Q.); 2 Key Laboratory of Crop Physiology, Ecology and Genetic Breeding, Ministry of Education, Key Laboratory of Chemical Utilization of Plant Resources of Nanchang, College of Chemistry and Materials, Jiangxi Agricultural University, Nanchang 330045, China; 18755870061@163.com (L.L.); 18370786250@163.com (X.L.); a845831015@163.com (L.D.); 3 Ji’an Key Laboratory of Photoelectric Crystal Materials and Device, Key Laboratory of Jiangxi Province for Special Optoelectronic Artificial Crystal Materials, School of Chemistry and Chemical Engineering, Humic Acid Utilization Engineering Research Center of Jiangxi Province, Institute of Applied Chemistry, Jinggangshan University, Ji’an 343009, China

**Keywords:** electrochemical sensor, heavy metal ions, Ni nanoparticles, MOF-derived carbon

## Abstract

Heavy metal pollution has posed a serious threat to the ecological environment and human health. Thus, the development of accurate and effective methods for their detection is crucial. In this study, a novel electrochemical sensor was fabricated to detect Cu^2+^ and Hg^2+^, based on N-doped carbon nanotube-wrapped Ni nanoparticle (Ni@N-CNT) sensing material, which was derived from the pyrolysis of Ni^2+^ doped ZIF-8. For electrode material design, the packaging structure not only protected the encapsulated Ni nanoparticles from electrochemical corrosion in the acid electrolyte but also provided excellent electro-catalytic activity and electrical conductivity by controlling their size. Thanks to the overall performance of the Ni@N-CNT composite, the proposed sensor exhibited excellent analytical performance for Cu^2+^ and Hg^2+^ detection, with ultra-low detection limits of 33.3 ng⋅L^−1^ and 33.3 ng⋅L^−1^, respectively. The sensor also demonstrated good repeatability, reproducibility and selectivity. In addition, the method was successfully applied to the electrochemical analysis of Cu^2+^ and Hg^2+^ in actual Chinese cabbage samples with satisfactory recovery, confirming its practical applicability.

## 1. Introduction

Heavy metal ion (HMI) pollution poses a tremendous threat to human health and ecological balance due to its high toxicity and nonbiodegradable nature [1,2,3]. Among heavy metal ions, Cu^2+^ and Hg^2+^ are recognized as the typical heavy metal contaminants [4,5,6]. Cu^2+^ is an indispensable element in biological metabolism [7], but excessive Cu^2+^ can lead to severe diseases and disorders, exhibiting toxicity comparable to that of Hg^2+^ [8]. Thus, there is an urgent need to develop rapid and sensitive analytical methods for the detection of Cu^2+^ and Hg^2+^ [9]. Electrochemical stripping voltammetry techniques have been widely regarded as powerful tools for HMI detection due to their inherent advantages of simple operation, fast reaction speed, low cost, and high selectivity [10,11,12]. In this context, the development of advanced functional electrode materials is of great importance to improve precision and sensitivity [13].

In recent years, metal–organic framework (MOF)-derived carbon materials have attracted significant attention in the field of electrocatalysis due to their unique properties, such as a well-defined porous structure, high electrical conductivity, abundant active sites and easy modification with other elements and materials [14,15,16]. Metal nanoparticle-embedded N-doped carbon nanotubes (N-CNTs) are especially promising because they enhance charge transfer efficiency, facilitate analyte diffusion and provide protection against harsh acidic/alkaline environments [17,18]. For example, Wang et al. developed N-CNT-encapsulated FeCoNiMnCr for high electrocatalytic activity toward the glucose oxidation and oxygen evolution reaction (OER) [19,20]. Dong et al. synthesized NiS nanocrystals capsulated by bamboo-like N-CNTs for the enhanced electro-oxidation of glucose [21,22]. Chen et al. produced a CoP nanoparticle-embedded N-CNT hollow polyhedron using ZIF-8@ZIF-67 for efficient overall water splitting [23]. These N-CNT encapsulation materials showed good applications in sensing. However, to the best of our knowledge, they have not yet been used for the simultaneous detection of Cu^2+^ and Hg^2+^.

**Scheme 1:**
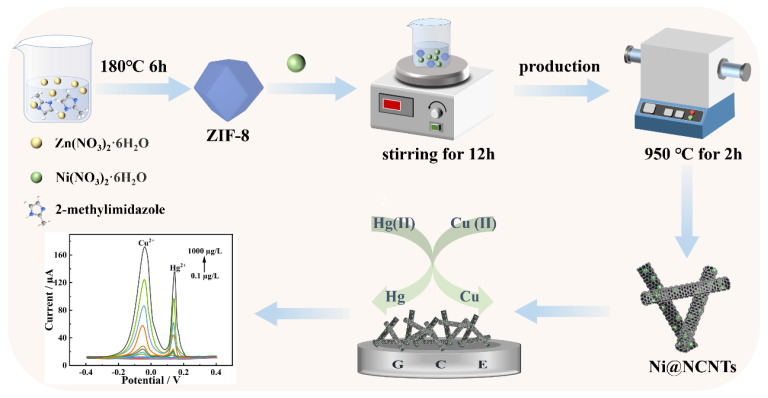
Schematic diagram of the preparation process of Ni@N-CNTs and their application for simultaneous detection of Cu^2+^ and Hg^2+^.

In this work, a facile two-step pyrolytic reduction strategy was proposed to prepare a N-doped carbon nanotube-encapsulated Ni nanoparticle (Ni@N-CNTs) hybrid using ZIF-8 as a precursor (Scheme 1). Dodecahedral ZIF-8 was first synthesized via the solvothermal method using 2-methylimidazole (2-MeIm) as a ligand and Zn ions as coordination centers. This was then mixed with Ni ions (Ni^2+^), and the resulting ZIF-8/Ni^2+^ complex was calcined to form Ni@N-CNTs.

In the synthetic system, the adsorbed metallic Ni acted as an efficient catalyst, promoting the conversion of carbon sources into thin-layer graphitic CNTs. The Ni nanoparticles were encapsulated within the porous bamboo-like N-CNTs, ensuring their electrocatalytic activity and stability. Meanwhile, the N-CNTs interweaved to form a conductive network that facilitates charge transport. The proposed Ni@N-CNT composite displayed excellent electrochemical performance for the detection of Cu^2+^ and Hg^2+^. In addition, the applicability of the Ni@N-CNT electrode was demonstrated by successfully detecting Cu^2+^ and Hg^2+^ in actual cabbage samples.

## 2. Results and Discussion

### 2.1. Morphological and Structural Characterization

**Figure 1:**
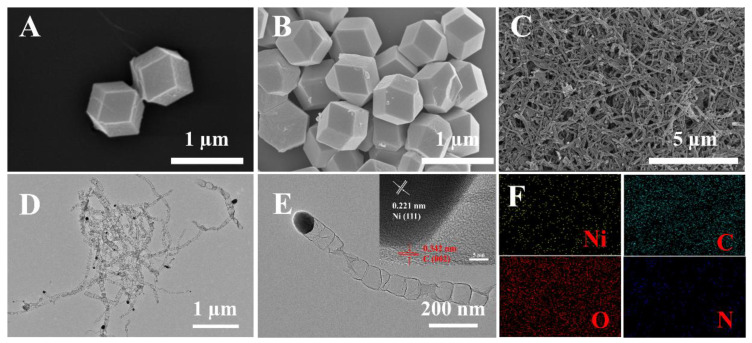
SEM images of (**A**) ZIF-8, (**B**) ZIF-8/Ni^2+^ and (**C**) Ni@N-CNTs, (**D**,**E**) TEM images of the Ni@N-CNTs and HR-TEM image of the Ni@N-CNTs, and (**F**) elemental mapping of Ni@N-CNTs.

The morphological details of ZIF-8, ZIF-8/Ni^2+^ and Ni@N-CNTs were characterized by SEM. As shown in Figure 1A, ZIF-8 presents a classic regular dodecahedron structure with a smooth surface. Figure 1B shows the SEM of ZIF-8/Ni^2+^. Ni doping had no significant effect on the morphology of ZIF-8, nor did the particle size change significantly. For Ni@N-CNTs (Figure 1C), the prepared sample displays a one-dimensional, bamboo-like structure with an average diameter of approximately 75 nm and a length of several micrometers. The morphology and micro-structure of the Ni@N-CNT composites were further studied via TEM. As shown in Figure 1D,E, it is evident that Ni nanoparticles are encapsulated in the tip of the bamboo-like, hollow-shaped and compartmentalized N-CNTs. From the high-resolution TEM (HRTEM) image of the Ni@N-CNTs (Figure 1E), the lattice spacings of 0.221 nm and 0.342 nm correspond to the (111) lattice plane of metallic Ni and the (002) lattice plane of graphitic carbon, respectively [24]. Additionally, energy dispersive X-ray spectroscopy (EDS) analysis (Figure 1F) confirms the coexistence of Ni, C, O and N elements in Ni@N-CNTs, further validating the presence of both Ni nanoparticles and N-CNTs in the composite. The introduction of N enhances the catalytic performance of Ni@N-CNTs.

**Figure 2:**
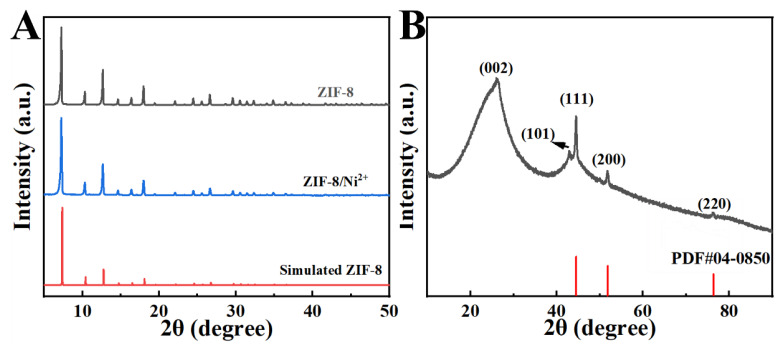
X-ray diffraction (XRD) patterns of ZIF-8 and ZIF-8/Ni^2+^ (**A**), Ni@N-CNTs (**B**).

X-ray diffraction (XRD) analysis was used to investigate the crystallographic structures of ZIF-8, ZIF-8/Ni^2+^ and Ni@N-CNTs. As shown in Figure 2A, the XRD pattern of ZIF-8 indicated that the main diffraction peaks were 7.43°, 10.45°, 12.68°, 14.75°, 16.45°, 18.02°, 19.45° and 22.21°, corresponding to the (110), (200), (211), (220), (310), (222), (321) and (330) crystal faces, respectively. The XRD patterns of the synthesized ZIF-8 basically matched with the simulated ZIF-8, confirming that the precursor of ZIF-8 was successfully prepared. For the ZIF-8/Ni²⁺ spectrum, the characteristic peaks were consistent with those of ZIF-8, indicating that the doping of Ni²⁺ did not change the crystal structure of ZIF-8. In Figure 2B, three distinct diffraction peaks were observed at 44.4°, 51.8° and 76.3°, corresponding to the (111), (200) and (220) crystal planes of metallic Ni (JCPDS 04-0850) [25]. These peaks confirm the presence of metallic Ni within the Ni@N-CNT composite. Notably, the Ni@N-CNT sample manifests two peaks at 25.3° and 44.2°, corresponding to disordered carbon (002) and graphitic carbon (101), respectively [26]. The presence of the (002) and (101) reflections suggests that the Ni@N-CNTs have a higher degree of graphitization. These results confirm the successful integration of Ni and N-CNTs in the composite.

**Figure 3:**
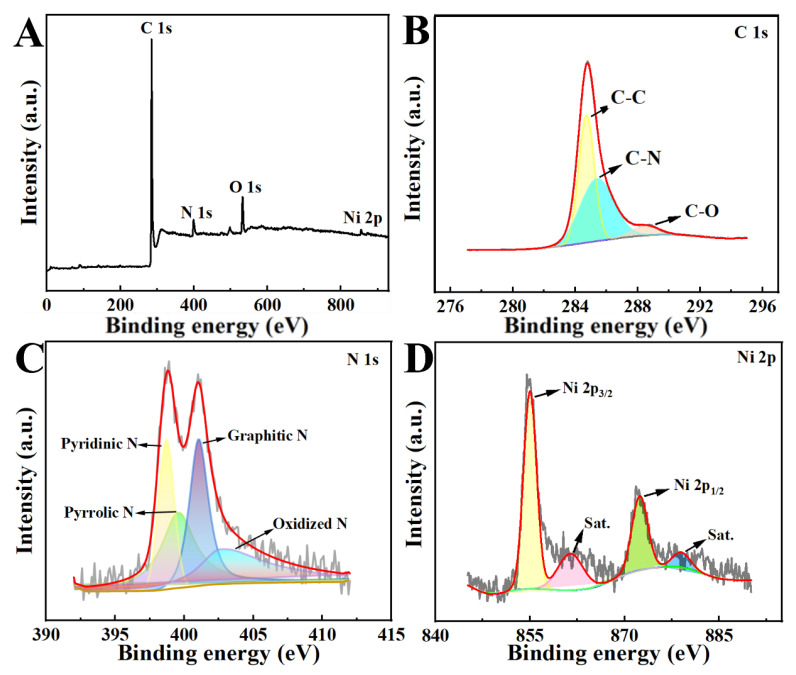
(**A**) Survey XPS spectrum of Ni@N-CNTs. High-resolution XPS spectra of (**B**) C 1s, (**C**) N 1s and (**D**) Ni 2p regions of the Ni@N-CNTs.

Furthermore, X-ray photoelectron spectroscopy (XPS) was utilized to investigate the chemical state and electronic structure of elements of the Ni@N-CNTs. As shown in Figure 3A, the XPS survey spectra confirm the presence of C, N, O and Ni elements. The O 1s peak, observed in the survey spectra of all samples, is attributed to adventitious oxygen on the surface of the material. The peak at ~500 eV is more likely associated with the O 1s satellite peak or a minor impurity from the sample preparation process, such as surface contamination or adventitious carbon species [27]. To further analyze the element state, the high-resolution spectra of each element were examined. The C 1s spectrum (Figure 3B) is deconvoluted into three peaks at 284.8 eV (C-C), 285.21 eV (C-N) and 289.31 eV (C-O) [28], indicating that N has been doped into the carbon skeleton. The N 1s spectrum in Figure 3C is decomposed into four peaks. The main forms of the N element are pyridine N (398.4 eV), Ni-N (399.3 eV), graphite N (401.2 eV) and a small amount of N oxide (404.3 eV) [29]. The variety of N species effectively regulates the electronic structure of carbon-based materials and enhances their hydrophilicity. In the Ni 2p XPS spectrum (Figure 3D), two prominent bands correspond to Ni 2p3/2 and 2p1/2 with binding energies at 855.7 eV (metallic Ni-Ni (0)) and 873.6 eV (Ni-N (II) oxidation state) and satellite peaks at 861.6 eV and 879.5 eV [30]. The presence of Ni-N bonds strengthens the coupling between Ni nanoparticles (NiNPs) and CNTs, fostering strong interactions between the active metal sites and the carbon nanotubes. The XPS characterization confirms the successful decoration of Ni on the N-CNTs.

**Figure 4:**
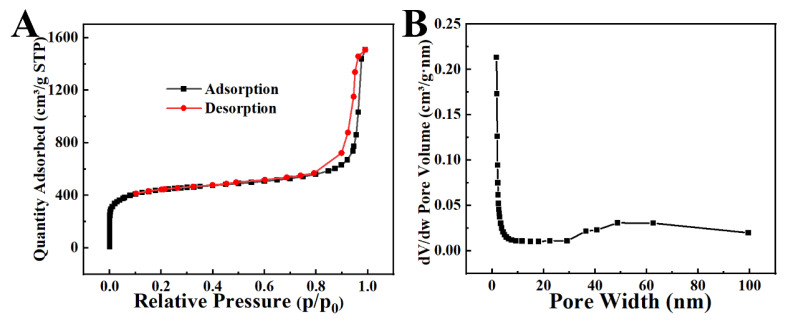
N_2_ adsorption–desorption isotherms of Ni@N-CNTs (**A**), pore size distribution of Ni@N-CNTs (**B**).

To evaluate the pore structure and specific surface area of the prepared nanocomposites, N_2_ adsorption/desorption isotherm tests were conducted at 77 K. Prior to the test, the sample was degassed at 300 °C for 6 h to ensure the removal of water and adsorption of impurities from the sample surface and pores. The N_2_ physisorption analysis (Figure 4A) reveals that Ni@N-CNT exhibits a type IV physisorption isotherm with a type H4 hysteresis loop, a distinctive characteristic of mesoporous materials [31]. In the low-pressure region (P/P_0_ < 0.05), Ni@N-CNTs show significant nitrogen uptake, resulting in a sharp increase in adsorption capacity. The Brunauer−Emmett−Teller (BET) analysis reveals that the Ni@N-CNT composite has a large specific surface area of 1652.67 m²·g^−1^. This large specific surface area provides ample accessible active sites and facile mass transport during electrocatalysis. The pore size distribution, shown in Figure 4B, indicates that the composite has a rich mesoporous structure, which enhances the enrichment of heavy metal ions.

### 2.2. Electrochemical Characterizations

**Figure 5:**
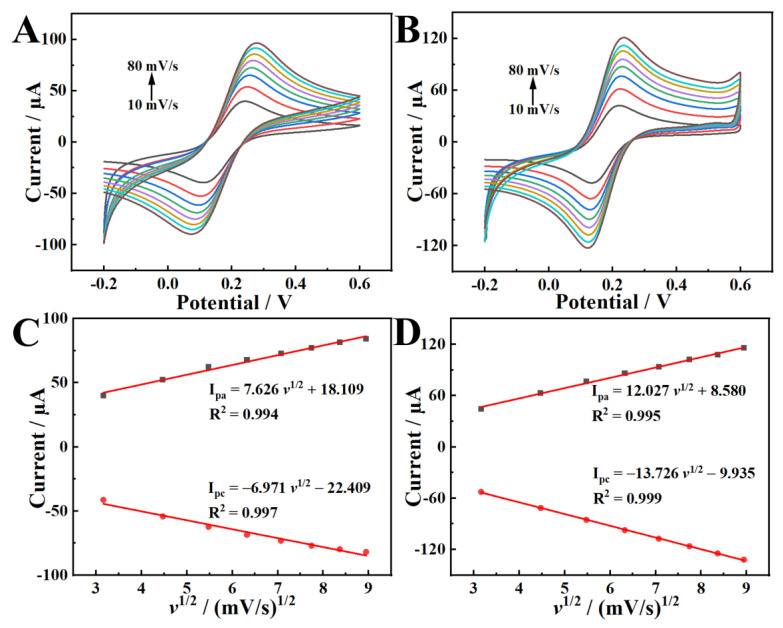
(**A**,**B**) CV curves of the bare GCE and Ni@N-CNTs/GCE in 5 mM [Fe(CN)_6_]^3−/4−^ containing 0.1 M KCl at different scan rates. (**C**,**D**) The corresponding linear relationship between the redox peak currents and the square root of scan rates for bare GCE and Ni@N-CNTs/GCE.

Cyclic voltammetry under different scan rates was measured to evaluate the analytical performance of the effective electroactive surface area (Aeff) of the various modified electrodes (Figure 5). Obviously, as the scanning rate increases, the peak current (Ip) of [Fe(CN)_6_]^3−/4−^ increases in the range of 10~80 mV·s^−1^ and is linearly proportional to the square root of the scan rate. The Aeff of the modified electrodes was calculated according to the Randles–Sevcik equation [32].

$$I_{P}=2.69\times 10^{5}\;n^{\frac{3}{2}}ACD^{\frac{1}{2}}v^{\frac{1}{2}}$$

where *I*_p_ represents the peak current, *n* denotes the electron transfer number (*n* = 1), *A* signifies the effective surface area, *C* refers to the concentration of the probe molecule in the redox solution (5.0 mM [Fe(CN)_6_]^3−/4−^), *D* is the diffusion coefficient (7.6 × 10^−6^ cm^2^·s^−1^), and *v* stands for the scan rate. Based on the fitting plot of *I*_p_ versus *ν*^1/2^, the Aeff of the bare GCE and Ni@N-CNTs/GCE is calculated to be 0.060 cm^2^ and 0.118 cm^2^, respectively. These results indicate that the Ni@N-CNTs/GCE has a superior electrolyte-electrode effective contact area.

**Figure 6:**
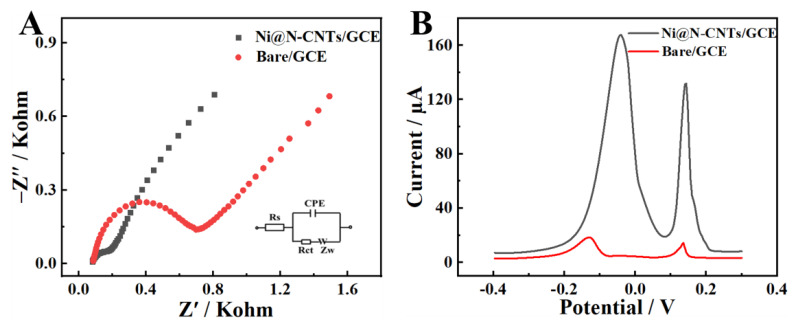
(**A**) Nyquist diagram for bare GCE and Ni@N-CNTs/GCE in 0.1 M KCl solution containing 5.0 mM [Fe(CN)_6_]^3−/4−^. (**B**) DPASV of 1 mg⋅L^−1^ Cu^2+^ and Hg^2+^ at the bare GCE and Ni@N-CNTs/GCE in 0.1 M ABS (pH = 4.5).

Furthermore, the charge transfer capabilities of different modified electrodes were investigated using electrochemical impedance spectroscopy (EIS) (Figure 6A). The Randles equivalent circuit was employed to simulate the data obtained from EIS measurements, where Rs, Ret, Zw and CPE represented solution resistance, electron transfer resistance, Warburg impedance and double layer capacitance, respectively [33]. Based on the equivalent circuit, the Ret value of the Ni@N-CNTs/GCE (84.37 Ω) was significantly lower than that of the bare GCE (516.2 Ω), indicating that the Ni@N-CNTs/GCE with excellent conductivity could accelerate the electron transfer on the electrode interface. These results suggest that the formation of N-CNTs enhances electrolyte permeability and effectively shortens the transport path for ions and electrons, thus promoting mass transfer.

The electrochemical behavior of Cu^2+^ (1.0 mg·L^−1^) and Hg^2+^ (1.0 mg·L^−1^) in 0.1 M ABS (pH = 4.5) at different modified electrodes was investigated by anodic dissolution voltammetry (DAPSV). As shown in Figure 6B, two relatively weaker dissolution peaks for Cu^2+^ and Hg^2+^ were observed under the bare GCE, while a more pronounced dissolution peak appears at the Ni@N-CNTs/GCE. This phenomenon can be attributed to the excellent enrichment ability of the large specific surface area of carbon tubes for Cu^2+^ and Hg^2+^, along with the synergistic effect between Ni and N-CNTs.

### 2.3. Experimental Parameters Optimization

**Figure 7:**
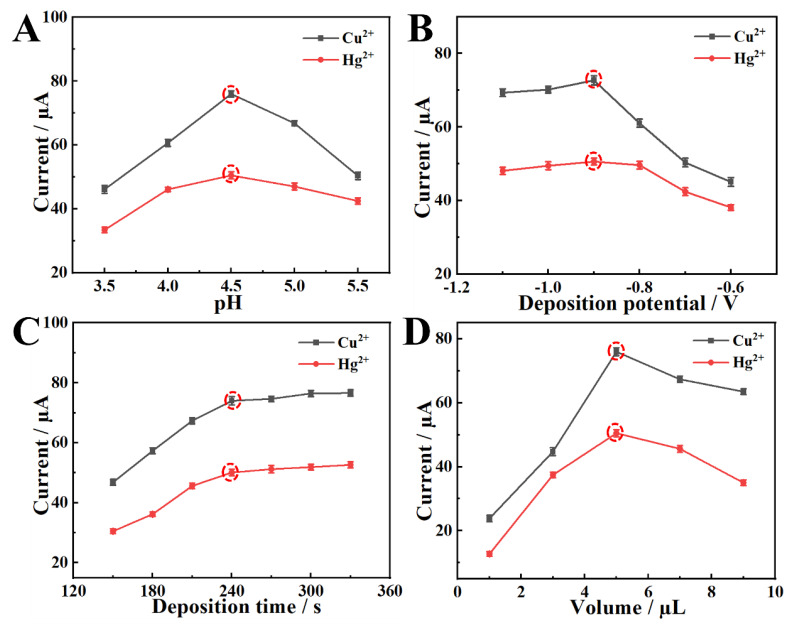
Optimization of experimental conditions. Effects of (**A**) pH of buffer solution, (**B**) deposition potential, (**C**) deposition time, and (**D**) Ni@N-CNT volume on the electrochemical responses of 0.5 mg·L^−1^ Cu^2+^ and Hg^2+^ at Ni@N-CNTs/GCE.

The effect of different pH values (ranging from 3.5 to 5.5) on the sensor performance toward 0.5 mg·L^−1^ Cu^2+^ and 0.5 mg·L^−1^ Hg^2+^ was investigated. As shown in Figure 7A, the current response gradually increased as the pH rose, peaking at pH 4.5. However, the current response was obviously reduced when the pH was over 4.5. This phenomenon is associated with the competitive reaction between H⁺ and Cu²⁺ and Hg²⁺ at lower pH values (from pH 3.5 to pH 4.0). At higher pH values (5.0 and 5.5), the formation of metal hydroxide species hindered adsorption and reduced current stripping [34]. Therefore, 4.5 was selected as the optimal pH for subsequent experiments.

The influence of deposition potential on response signals is examined in Figure 7B. It reveals that when the deposition potential decreased from −0.6 V to −0.9 V, the peak currents of Cu^2+^ and Hg^2+^ gradually increased. This increase is attributed to the negative shift in deposition potential, which facilitates further reduction of the metal ions. However, as the deposition potential shifted further negatively from −0.9 V to −1.1 V, the dissolution peak currents of both Cu^2+^ and Hg^2+^ decreased. This decrease is due to the onset of the hydrogen evolution reaction at more negative deposition potentials, which leads to the formation of H₂ bubbles that interfere with the electrodeposition process [35]. Thus, −0.9 V was selected as the optimal deposition potential.

Figure 7C depicts the effect of deposition time on the current response. The peak currents of Cu^2+^ and Hg^2+^ gradually increased with increasing deposition time from 150 to 240 s. However, when the deposition time exceeded 240 s, the current signal basically remained relatively unchanged. This suggests that the enrichment of metal ions on the working electrode gradually becomes saturated [36]. Therefore, 240 s was selected as the optimal deposition time.

As demonstrated in Figure 7D, the peak currents of Cu^2+^ and Hg^2+^ significantly increased with the increase in Ni@N-CNT drip volume from 1 to 5 µL. This increase is likely due to the greater number of active sites. However, a further increment in drop volume resulted in a decreased peak current. This phenomenon may be attributed to electron transfer caused by an excessive volume of Ni@N-CNTs. Consequently, 5 µL was selected as the optimal volume for electrode modification.

### 2.4. DPASV Detection of Cu^2+^ and Hg^2+^

**Figure 8:**
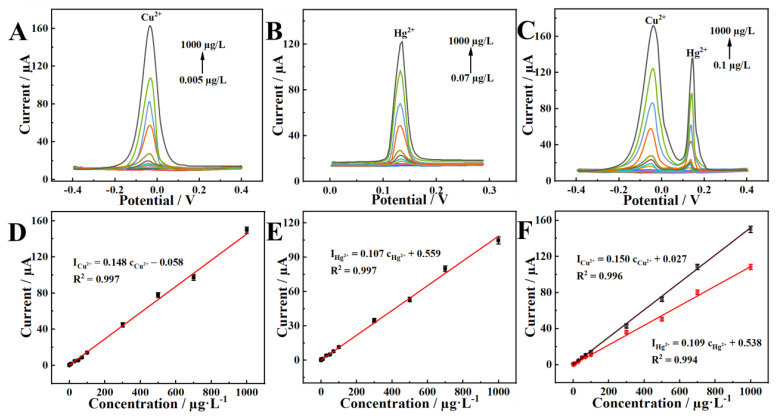
DPASV responses of the Ni@N-CNTs/GCE for the individual analysis of (**A**) Cu^2+^ over a concentration range of 0.005 µg·L^−1^ to 1000 µg·L^−1^, (**B**) Hg^2+^ over a concentration range of 0.07 µg·L^−1^ to 1000 µg·L^−1^ and (**C**) Cu^2+^ and Hg^2+^ over a concentration range of 0.1 µg·L^−1^ to 1000 µg·L^−1^. The corresponding linear relationships between current and concentration of the target heavy metal: (**D**) Cu^2+^, (**E**) Hg^2+^ and (**F**) Cu^2+^ and Hg^2+^. Different colored lines represent different concentrations of heavy metal ions.

Differential pulse anodic stripping voltammetry (DPASV) is a powerful electrochemical technique for detecting heavy metal ions in aqueous samples, involving two key processes: preconcentration and stripping. In the preconcentration step, a negative potential is applied to the working electrode, driving the deposition of target heavy metal ions onto its surface. This process is influenced by factors such as deposition time and applied potential. During the stripping stage, the electrode potential is scanned in the positive direction, causing the deposited metal ions to be re-oxidized and stripped off the electrode. This generates a current that is directly proportional to the concentration of the metal ions. The resulting peak currents can be used for quantitative analysis and differentiation of heavy metals, based on their unique stripping potentials. Under the optimized experimental conditions, DPASV was employed for the individual determination of Cu^2+^ and Hg^2+^ at the Ni@N-CNTs/GCE. As shown in Figure 8A, the peak current at approximately −0.05 V gradually increased upon the continuous addition of Cu^2+^ to the test solution. Moreover, the peak current increased proportionally with the Cu^2+^ concentration over the range of 0.005 to 1000.0 µg L^−1^. The corresponding linear regression equation was I_Cu_^2+^ = 0.148 c_Cu_^2+^ −0.058, with a correlation coefficient (R^2^) of 0.9970 (Figure 8D). The low limit of detection (LOD) for Cu^2+^ was determined to be 1.67 ng L^−1^, based on a signal-to-noise ratio (S/N) of 3. Similarly, the Ni@N-CNTs/GCE was also utilized for the detection of Hg^2+^. As depicted in Figure 8B, Hg^2+^ was distinctly identified at a potential of 0.13 V. The stripping current increased linearly with the Hg^2+^ concentration across the range of 0.07 to 1000.0 µg L^−1^. The linear equation for Hg^2+^ was I_Hg_^2+^ = 0.107 c_Hg_^2+^ + 0.559, with a correlation coefficient (R^2^) of 0.9970 and an LOD of 23.3 ng L^−1^ (Figure 8E).

Furthermore, the Ni@N-CNTs/GCE was employed for the simultaneous detection of Cu^2+^ and Hg^2+^ in a 0.1 M ABS with a pH of 4.5, as shown in Figure 8C. The peak response currents were positively correlated with the concentrations of Cu^2+^ and Hg^2+^ over the range of 0.1 µg L^−1^ to 1000.0 µg L^−1^ (Figure 8F). The corresponding linear regression equations were I_Cu_^2+^ = 0.150 c_Cu_^2+^ + 0.027 (R^2^ = 0.996) for Cu^2+^ and I_Hg_^2+^ = 0.109 c_Hg_^2+^ + 0.538 (R^2^ = 0.994) for Hg^2+^. The LODs for Cu^2+^ and Hg^2+^ were both calculated to be 33.3 ng L^−1^ (S/N = 3). These results demonstrate that the constructed electrochemical platform exhibits high sensitivity and the ability to simultaneously detect multiple HMIs. In comparison with some previous reports (Table 1), it shows satisfactory LODs. These attractive features are attributed to several aspects. On the one hand, the Ni@N-CNT composite exhibits a porous network structure, which facilitates sufficient contact between the target HMIs and the electrode, thus significantly improving mass transport efficiency. On the other hand, the NiNPs and N-CNTs provide excellent electron conductivity and abundant active sites, enhancing electrochemical performance.

### 2.5. Repeatability, Reproducibility, Stability and Selectivity of Ni@N-CNTs/GCE

**Figure 9:**
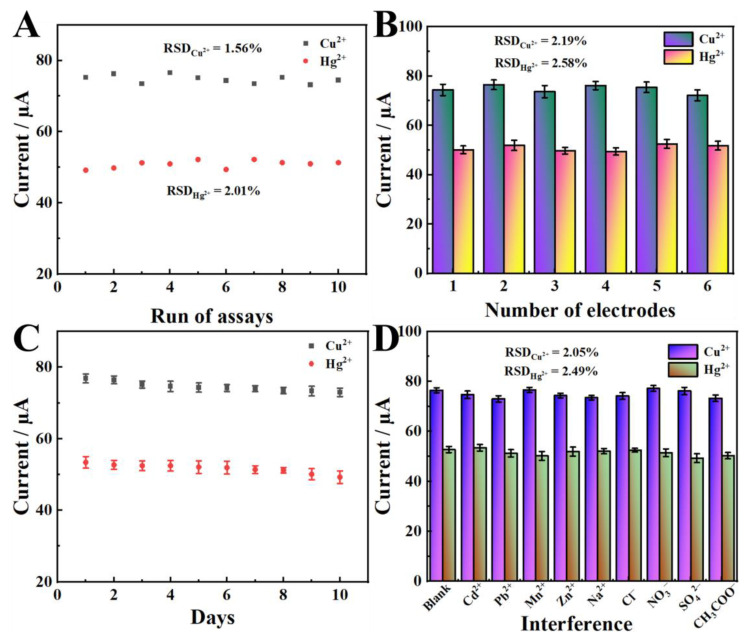
(**A**) Repeatability measurements for one Ni@N-CNTs/GCE, (**B**) reproducibility measurements at six independent Ni@N-CNTs/GCEs, (**C**) stability measurements of Ni@N-CNTs/GCE within 10 days and (**D**) anti-interference ability of Ni@N-CNTs/GCE.

The repeatability of the constructed sensor for 0.5 mg L^−1^ Cu^2+^ and 0.5 mg L^−1^ Hg^2+^ detection was verified by ten successive measurements. As shown in Figure 9A, the RSDs of the response current are 1.56% for Cu^2+^ and 2.01% for Hg^2+^, respectively, indicating satisfactory repeatability. In addition, six parallel Ni@N-CNTs/GCEs were prepared to evaluate the reproducibility of the proposed sensor (Figure 9B). The RSDs for Cu^2+^ and Hg^2+^ were 2.19% and 2.58%, respectively. These results demonstrate that the modified electrode possesses commendable repeatability and reproducibility.

The stability of the Ni@N-CNTs/GCE sensor was evaluated by monitoring 0.5 mg L^−1^ Cu^2+^ and 0.5 mg L^−1^ Hg^2+^ over ten consecutive days. As depicted in Figure 9C, the current response of the Ni@N-CNTs/GCE exhibits only a slight decrease, retaining 94.93% and 92.27% of its initial response to Hg^2+^ and Cu^2+^, respectively. The superior stability of the Ni@N-CNTs/GCE is attributed to the protective carbon nanotube shell, which shields the highly active NiNPs from electrochemical corrosion by the electrolyte, thereby enhancing the overall stability of the sensor.

The selectivity of Ni@N-CNTs/GCE for the electrochemical mensuration of 0.5 mg⋅L^−1^ Cu^2+^ and 0.5 mg⋅L^−1^ Hg^2+^ was investigated in a mixed solution containing some potential interfering ions (50-fold Cd^2+^, Pb^2+^, Mn^2+^, Zn^2+^, Na^+^, Cl^−^, NO_3_^−^, SO_4_^2−^ and CH_3_COO^−^). The change in the current response with and without the interferents was recorded. As shown in Figure 9D, all the current responses were altered by less than 5%. These results demonstrate that the developed Ni@N-CNTs/GCE exhibits excellent anti-interference and selectivity.

### 2.6. Real Sample Analysis

The practicability of Ni@N-CNTs was assessed for quantifying Cu^2+^ and Hg^2+^ levels in cabbage samples. The pretreatment of the cabbage samples is described in Section 3.6 and the content of Cu^2+^ and Hg^2+^ in actual samples was determined using the standard addition method. As shown in Table 2, the acceptable recoveries ranged from 98.0% to 103.0% for Cu^2+^ and 99.0% to 102.8% for Hg^2+^. Additionally, all RSDs of the recoveries were less than 2.14%, demonstrating that the Ni@N-CNTs/GCE exhibited reliable and accurate performance for determining Cu²⁺ and Hg²⁺ in real samples.

## 3. Experimental Section

### 3.1. Materials and Reagents

N, N-dimethylformamide (DMF, 99.5% purity), zinc nitrate hexahydrate (Zn(NO_3_)_2_·6H_2_O, 99% purity), nickel nitrate hexahydrate (Ni(NO_3_)_2_·6H_2_O, 98% purity), 2-methylimidazole (2-MeIm, 98% purity), methanol (CH_3_OH, 99.5% purity), ethyl alcohol (CH_3_CH_2_OH, 99.7% purity), acetic acid (CH_3_COOH, 99.5% purity), sodium acetate (CH_3_COONa, 99% purity), potassium ferricyanide (K_3_Fe(CN)_6_, 99.5% purity), potassium ferrocyanide (K_4_Fe(CN)_6_, 99.5% purity) and potassium chloride (KCl, 99.5% purity) were purchased from Aladdin BioChem Technology Co., Ltd. (Shanghai, China). All solutions in this experiment were prepared using ultrapure water with an electrical resistivity of at least 18.25 MΩ cm^−1^, which was acquired via the Milli-Q water purification system (Billerica, MA, U.S.). All chemicals obtained in this work were analytical grade and were used without any pre-treatment.

### 3.2. Apparatus

The composition and crystal structure of the samples were analyzed using powder X-ray diffraction (PXRD), which was carried out on a Bruker D8 Advance MKII machine equipped with a Cu K alpha-ray source (Eindhoven, Netherlands). The morphology and energy dispersive spectroscopy (EDS) of the samples were characterized using an S-4800 cold field emission scanning electron microscope (SEM) (Hitachi, Tokyo, Japan). Transmission electron microscope (TEM) images were studied using an American-made G2 T20 transmission electron microscope at an accelerated voltage of 300 Kv (Thermo Fisher Scientific, Waltham, U.S.). X-ray photoelectron spectroscopy (XPS) analysis was conducted using an X-ray photoelectron spectroscope (Thermo Fisher Scientific, Waltham, U.S.). The Brunauer–Emmett–Teller (BET) specific surface area and porous structure were measured at 77 K by a Micromeritics ASAP 2020 system (Micromeritics, Norcross, U.S.). All electrochemical experiments were performed on a CHI760E (CH Instruments, Shanghai, China) workstation using a conventional three-electrode system, consisting of a saturated calomel electrode, a platinum wire electrode and a glassy carbon electrode (GCE, *Φ* = 3 mm).

### 3.3. Synthesis of Ni@N-CNTs

Firstly, Zn(NO_3_)_2_·6H_2_O (3.2 mmol) and 2-MeIm (12.8 mmol) were dissolved in 35 mL of CH_3_OH, respectively. The above solutions were mixed and transferred to a 100 mL Teflon-lined, stainless-steel autoclave, where the reaction was carried out at 180 °C for 6 h. After that, the products were collected through centrifugation (6000 rpm, 3 min) and washed three times with methanol. The obtained ZIF-8 was dried overnight at 70 °C for subsequent use.

In order to synthesize the Ni@N-CNT composite, a ZIF-8/Ni^2+^ complex was first prepared. Specifically, 0.3 g of ZIF-8 was dispersed in 30 mL of DMF, and 0.3 g of Ni(NO_3_)_2_·6H_2_O in 50 mL of DMF was slowly added to the solution while stirring continuously for 12 h. The mixture was centrifuged, washed alternately with ethanol and ultrapure water three times and dried overnight at 70 °C. The resulting sample was labeled ZIF-8/Ni^2+^. Finally, the dried precursor of ZIF-8/Ni^2+^ was sintered in a nitrogen (N_2_) atmosphere, heated at 5 °C min^−1^ from room temperature to 950 °C and held at this temperature for 2 h to obtain Ni@N-CNTs. The synthesis process of Ni@N-CNTs is illustrated in Scheme 1.

### 3.4. Preparation of Ni@N-CNT-Modified Electrode

First, 3 mg of Ni@N-CNTs were dispersed in 1 mL of ultrapure water to prepare a 3 mg·mL^−1^ Ni@N-CNT suspension with the aid of ultrasonication. Prior to modification, the bare GCE was polished on the suede surface with 0.05 µm alumina powder and then washed in an ultrasonic cleaner with ethanol and ultrapure water. Subsequently, 5 μL of the 3 mg·mL^−1^ Ni@N-CNT dispersion was drop-cast onto the surface of the freshly polished GCE and dried in an oven at 45 °C to obtain the Ni@N-CNTs/GCE.

### 3.5. Electrochemical Measurements

Electrochemical impedance spectroscopy (EIS) was utilized for the electrochemical characterization of the various modified electrodes in an electrolyte solution containing 5 mM [Fe(CN)_6_]^3−/4−^ and 0.1 M KCl. The frequency range for EIS measurements was from 1 Hz to 10^5^ Hz with an amplitude of 5 mV. Cyclic voltammetry (CV) experiments were carried out in a 0.1 M KCl solution containing 5 mM [Fe(CN)_6_]^3−/4−^, with a potential range from—0.2 V to 0.6 V. DPASV was carried out for the detection of Hg^2+^ and Cu^2+^. Briefly, the modified electrode was immersed in 0.1 M acetate buffer solution (ABS) containing a specific concentration of Hg^2+^ and Cu^2+^. Accumulation of the metal ions on the electrodes was acquired at a deposition potential of—0.9 V for 240 s.

### 3.6. Preparation of Real Samples

Cabbage was purchased from a local supermarket. After being mashed in a mortar, the sample was filtered with a 0.45 µm filter membrane. The collected filtrate was then centrifuged at 8000 rpm for 5 min to obtain the clarified supernatant. The supernatant was diluted 50 times with 0.1 M acetate buffer solution (ABS) (pH = 4.5) to prepare the real sample for analysis.

## 4. Conclusions

In this work, high-quality Ni nanoparticles embedded in N-doped carbon nanotubes (Ni@N-CNTs) were successfully prepared by a facile two-step strategy with ZIF-8 as the precursor. Given that each heavy metal ion has a unique redox potential, with the peak position of Cu²⁺ being approximately -0.05 V and that of Hg²⁺ around 0.13 V, an electrochemical sensing platform was constructed using Ni@N-CNTs to enable simultaneous detection of Cu²⁺ and Hg²⁺. This design takes advantage of the unique electrochemical properties of these ions to achieve selective and accurate determination. The Ni@N-CNTs were found to enhance electron conduction, exposing more active sites and preventing electrochemical corrosion of Ni nanoparticles in acid media, thus improving electrochemical stability. Benefiting from the synergistic effect between the active metal and the carbon carrier, the Ni@N-CNTs/GCE demonstrated excellent performance for simultaneously detecting Cu^2+^ and Hg^2+^, with an ultra-low LOD of 33.3 ng⋅L^−1^ for both ions. Furthermore, the Ni@N-CNTs/GCE exhibited good repeatability, reproducibility and selectivity. This work could provide valuable insights into monitoring heavy metal ions in the natural environment and food.

## Figures and Tables

**Table 1:** Performance comparison with previously reported sensors for electrochemical detection of Hg^2+^ and Cu^2+^.

Electrode	Method	Linear Range (μg⋅L^−1^)		LOD (μg⋅L^−1^)		Reference
		Cu^2+^	Hg^2+^	Cu^2+^	Hg^2+^	
L-cys-rGO	DPASV	25.42–127.09	80.24–401.18	0.26	1.11	[37]
Layered N-doped carbon/MXene	SWASV	31.76–635.50	200.59–2005.90	1.25	11.23	[38]
NH2-CMS	DPASV	25.42–76.26	80.24–240.71	0.70	19.66	[39]
N,P- Ti_3_C_2_T_x_R	DPV	1.27–635.50	10.03–501.48	1.14	0.06	[40]
3D MXene-AuNPs@MBA (EAMTD/TGA)	DPASV	1–1300	1–1300	0.13	0.21	[41]
Ni@N-CNTs	DPASV	0.1–1000	0.1–1000	0.03	0.03	This work

**Table 2:** Recoveries and RSD obtained from practical samples spiked with Cu^2+^ and Hg^2+^ (*n* = 3).

Sample	Added (µg·L^−1^)		Found (µg·L^−1^)		Recovery (%)		RSD (%)	
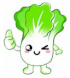 Cabbage	Cu^2+^	Hg^2+^	Cu^2+^	Hg^2+^	Cu^2+^	Hg^2+^	Cu^2+^	Hg^2+^
	0	0	0	0	-	-	-	-
	0.1	0.1	0.098	0.101	98.0	101.0	2.14	2.07
	1	1	1.03	0.99	103.0	99.0	1.87	1.73
	10	10	9.87	10.28	98.70	102.80	2.01	2.06
	100	100	100.43	102.17	100.40	102.20	1.37	1.79

## Data Availability

The data presented in this study are available in the article.
